# *Allium mongolicum* Regel Ethanol Extract Remodels Plasma Metabolome and Lipid Metabolism While Modulating Milk Metabolite Profiles in Dairy Cows

**DOI:** 10.3390/ani16081191

**Published:** 2026-04-14

**Authors:** Chen Bai, Xiaoyuan Wang, Guoli Han, Qina Cao, Yankai Zheng, Jiayu Duan, Huabei Li, Changjin Ao, Khas Erdene

**Affiliations:** Key Laboratory of Animal Nutrition and Feed Science at Universities of Inner Mongolia Autonomous Region, College of Animal Science, Inner Mongolia Agricultural University, Zhaowuda Road 306, Saihan District, Hohhot 010008, China

**Keywords:** *Allium mongolicum* Regel, blood, milk, fatty acids, metabolomics, cow

## Abstract

Blood metabolism in dairy cows is crucial for milk quality, functioning primarily through the “blood–milk” metabolic axis. *Allium mongolicum* Regel (AMR), a functional *Allium* herb, has been shown to regulate on ruminant lipid metabolism. This study investigated the impact of AMR ethanol extract (AME) on lactation performance, blood lipid parameters, and blood–milk metabolomes. Twelve mid-lactation Holsteins (606 ± 11 kg; milk yield 33.14 ± 2.08 kg/d) of parity 2–3 were assigned to either a basal diet (**CON**) or a diet supplemented with 54 g/d of AME (**AEE**). Results indicated that AME significantly decreased plasma triglycerides (TG), C15:0, C16:1, C18:1 n-9 c, C18:3 n-6, monounsaturated fatty acids (*p* < 0.05) and significantly increased C18:2 n-6 c, polyunsaturated fatty acids (*p* < 0.05). Lactation performance, including the average daily dry matter intake, daily yields of milk fat, protein and lactose, remained unaffected by the AME addition (*p* > 0.05). Metabolomic profiling revealed that AME significantly enriched the glycerophospholipid metabolism pathway in plasma, upregulating key phospholipid precursors such as L-serine and Sphinganine. Concurrently, milk metabolomics showed an upregulation of short-chain Acylcarnitines. Plasma TG correlated negatively with both plasma L-serine and milk Acylcarnitines, whereas low-density lipoprotein correlated positively with these energy-driven milk metabolites. These findings suggest that AME may contribute to remodeling the plasma lipid metabolic profile in a manner that could facilitate plasma-to-milk lipid flux. This appears to occur through enhanced hepatic lipid processing and increased mammary lipid utilization, offering preliminary insights into potential nutritional strategies for supporting lipid metabolism in dairy cows.

## Simple Summary

What a dairy cow consumes and how its body processes nutrients directly affect the nutritional quality of its milk. *Allium mongolicum* Regel, a type of functional wild onion, is known to positively influence animals’ fat metabolism. In this study, we investigated whether feeding an extract from this plant to lactating dairy cows could improve both their metabolic status and the composition of their milk. We fed one group of dairy cows exclusively a basal ration, and a second set was given the same feed supplemented with *Allium mongolicum* Regel extract. We then conducted a detailed analysis of their blood and milk components. We found that the extract did not negatively affect the cows’ milk yield. Instead, it successfully lowered certain undesirable fats in their bloodstream while showing the potential to increase beneficial fats. Furthermore, by analyzing both blood and milk, we discovered that *Allium mongolicum* Regel extract helps the liver process fats more efficiently and encourages the udder to utilize those circulating fats for energy. Blood lipids, specifically triglycerides and LDL cholesterol, may act a physical bridge connecting these two processes. Ultimately, *Allium mongolicum* Regel extract creates an optimized “blood-to-milk” fat flow. This prevents fat buildup in the liver and partitions more energy toward milk synthesis, offering a promising nutritional strategy to support the metabolic health of lactating dairy cows.

## 1. Introduction

Certain natural plant extracts can increase the abundance of beneficial compounds, including specific lipids alongside antioxidant molecules, within milk, which is vital for enhancing the nutritional value of dairy products [1,2,3]. The milk metabolome is highly dependent on maternal systemic blood metabolism, operating via the “blood–milk” metabolic axis [2]. Small-molecule metabolites in the blood enter the mammary gland via transmembrane transport or receptor-mediated pathways to be directly or indirectly involved in the remodeling of the milk metabolome. Furthermore, certain metabolites can act as signaling molecules that regulate energy reallocation and synthetic cascades within mammary epithelial cells [4]. Consequently, the composition, concentration, and binding state of blood metabolites directly dictate changes in the milk metabolic profile. Recent studies highlight that supplementing dairy cow diets with plant extracts rich in polyphenols and flavonoids, such as red clover, rosemary, and citrus peel extracts, can reshape systemic metabolic networks, particularly protein and lipid metabolism processes [2,5,6]. This, in turn, drives the targeted enrichment and transformation of milk metabolites [5,7]. However, the precise mechanisms by which specific bioactive compounds regulate the flux of key lipid precursors across the blood–mammary barrier remain poorly understood.

As a functional plant species from the *Allium* family, *Allium mongolicum* Regel (AMR) is native to regions such as Inner Mongolia, Gansu, and Ningxia in China [8]. Compared with red clover, rosemary, and citrus peel extracts, which are primarily rich in specific isoflavones or phenolic diterpenes [2,5,6], AMR is distinguished by a more diverse composition. In addition to being rich in flavonoids, AMR contains significantly higher levels of organic acids, nucleotides, and amino acids [9], suggesting that its regulatory role in the lipid flux from the rumen to the mammary gland may be more complex and multifaceted. Our previous studies established that dietary supplementation with AMR extract profoundly alters systemic lipid metabolism and hormone secretion profiles in meat sheep [10,11]. It also regulates lipid-related gene expression [12], methylation patterns, and fatty acid composition within adipose tissues [13,14,15]. Furthermore, we found that AMR modulates populations of rumen microbes responsible for biohydrogenation within lactating cattle [16]. Given this profound dependence of the mammary gland on blood-derived lipid precursors for milk synthesis, we postulate that the bioactive components of AMR, upon absorption into the bloodstream, trigger shifts in systemic lipid metabolism. These systemic shifts likely serve as upstream drivers to targeted reprogramming of the mammary lipid metabolism, thereby altering the milk metabolite composition.

We hypothesized that dietary supplementation with AMR ethanol extract (AME) reshapes systemic blood metabolism in dairy cows, thereby altering the precursor microenvironment for the mammary gland and driving reconstruction of the milk metabolome. To test this hypothesis, we evaluated the effects of AME on lactation performance, plasma lipid profiles, and mammary metabolism using integrated untargeted metabolomics of blood and milk. This approach aimed to clarify the blood-to-milk metabolic cascade and provide mechanistic insights into nutritional strategies for high-quality milk production.

## 2. Materials and Methods

### 2.1. Ethics Approval

The current study was ethically cleared by the Animal Welfare and Ethics Committee at Inner Mongolia Agricultural University on 6 March 2023 (NND2023016).

### 2.2. Preparation of AME

Fresh AMR materials were procured within Alxa Left Banner, Inner Mongolia, China. It was then dried at 65 °C and pulverized for later use. Briefly, AME was obtained using an ultrasonic extraction process. Concentration of the extract was achieved via vacuum evaporation using 75% ethanol conditions, and the concentrate was prepared into a lyophilized powder. The specific extraction procedure referred to the optimized method for preparing AME by Wang et al. [16]. The yield of the AME was 28%. Its main active components and their contents were: flavonoids (26.43%), organic acids including related derivatives (18.57%), nucleotides along with related derivatives (14.43%), amino acids (11.14%), vitamins (1.55%), choline (1.00%), lipids (7.22%), plus others [9].

### 2.3. Experimental Design and Animal Care

The research was carried out at an operational dairy facility in Hangjin Houqi, Bayannur City, Inner Mongolia, China. Twelve healthy mid-lactation Holstein cows (DIM = 123 ± 9 d) were enrolled, characterized by 2–3 parities, comparable body condition, a milk yield of 33.14 ± 2.08 kg/d, and initial body weight of 606 ± 11 kg. The experiment adopted a single-factor completely randomized design, with six replicates per group (one cow per replicate). Cows were kept in separate pens. Animals in the control group (**CON**) were given a standard TMR, whereas those in the treatment group (**AEE**) consumed the identical TMR enriched with 54 g/d AME per animal each day. The supplementation dose of AME was determined based on a previous in vitro study as well as the measured dry matter intake of the dairy cows during the pre-feeding period in the present study [16]. To ensure precise and complete ingestion, the extract was administered via oral gavage, another report from this study on serum immune and antioxidant indices corroborated that the oral drenching method did not induce a stress response in dairy cows [17].

The trial spanned a total of 42 d, which included a 14-day acclimation phase prior to a 28-day formal testing stage. Feed was dispensed daily at 02:00 and 14:00, and feed refusals were weighed daily to monitor intake. Milk collection occurred thrice daily at 01:30, 09:00, alongside 16:00. Throughout the study, cows were provided free access to unlimited feed and fresh water, maintaining sanitary living conditions. Details regarding the formula and nutritional makeup of the standard TMR can be found in Table 1.

### 2.4. Sample Collection

On day 28 of the experimental period, blood samples (40 mL) were collected from the jugular vein of each animal between 06:00 and 06:30, following overnight fasting with free access to water. The samples were collected into tubes containing EDTA and subsequently centrifuged at 12,000× *g* for 10 min. The resulting plasma was decanted into 1.5 mL tubes and stored at −20 °C for further analysis. On the same day, milk samples were gathered thrice daily (01:30, 09:00, and 16:00) and combined in a 4:3:3 proportion. The composite milk samples were preserved at −20 °C prior to testing.

### 2.5. Assessing Plasma Lipid Profiles

We measured plasma concentrations of several markers, including triglycerides (TG), cholesterol (CHOL), alongside low- and high-density lipoprotein cholesterol (LDL-C, HDL-C), plus free fatty acids (FFA) in blood samples. All necessary diagnostic kits were sourced via Beijing Lepu Bio-Technology Co., Ltd. (Beijing, China). A Hitachi 3110 automatic biochemical analyzer (Hitachi, Ltd., Tokyo, Japan) was used for the measurements.

### 2.6. Measurement of Average Daily Dry Matter Intake, Milk Yield and Composition

Milk yield was recorded using a 24-stall automated milking system and according to Duan et al. [18]. In this study, average daily dry matter intake (ADMI), daily yields of milk fat (DMF), daily production of protein (DMP), alongside lactose (DML), was determined using raw data from Duan et al. [18], using the following equations:

(1) $$\mathrm{ADMI}\;(\mathrm{kg}/d)\;=\;\frac{\mathrm{Total}\;\mathrm{dry}\;\mathrm{matter}\;\mathrm{intake}}{\mathrm{Trial}\;\mathrm{days}}$$

DMF (kg/d) = milk yield × milk fat percentage (2)

DMP (kg/d) = milk yield × milk protein percentage (3)

DML (kg/d) = milk yield × milk lactose percentage(4)

### 2.7. Determination of Plasma Fatty Acid Profile

For the analysis of plasma fatty acid profiles, total lipids were extracted from approximately 200 µL of plasma. The extraction process rigorously followed the established standard chloroform-methanol procedure (2:1, *v*/*v*) described by Folch et al. [19]. Following solvent vaporization beneath a nitrogen stream and the lipid residue weighed, the isolated lipids underwent a two-step transmethylation procedure to convert them into fatty acid methyl esters (FAMEs). In brief, approximately 20 mg of isolated lipids underwent saponification via dissolution within 2 mL of 0.5 M methanolic NaOH (80 °C, 15 min). After cooling, 2 mL of 14% BF_3_-methanol facilitated methylation during a subsequent 5 min incubation at 80 °C. We subsequently extracted the generated FAMEs using 2 mL of n-hexane, maintaining Nonadecanoic acid (C19:0) as the internal reference compound throughout this procedure.

FAME analysis was subsequently performed using gas chromatography (450GC, Varian Inc., Palo Alto, CA, USA) configured with both a flame ionization detector alongside a Supelco fused silica capillary column (SP-2560, 100 m × 0.25 mm × 0.20 μm; Bellefonte, PA, USA). Both detector and injector ports were set to 260 °C. The temperature thermal cycle commenced at 120 °C (maintained 5 min), followed by a 3 °C/min ramp to 230 °C (3 min hold). Subsequent heating phases included reaching 240 °C (1.5 °C/min ramp, 13 min hold), finally peaking at 245 °C (20 °C/min ramp, 6 min hold). Nitrogen acted as our carrier medium. The split ratio was set as 1:40. Individual fatty acids were characterized through alignment of their retention times against components within a purchased FAME reference blend (Supelco 37 Component FAME Mix, Sigma-Aldrich, St. Louis, MO, USA).

### 2.8. Milk and Plasma Metabolomic Profiling

To isolate metabolites, a 100 μL aliquot from each milk or plasma specimen was pipetted into a 1.5 mL tube, followed by the addition of 400 μL chilled extraction solvent (acetonitrile/methanol, 1:1, *v*/*v*), spiked with 0.02 mg/mL L-2-chlorophenylalanine serving as the internal reference. Following 30 s of vortex mixing, samples underwent sonication (5 °C, 40 KHz, 30 min). To induce protein precipitation, this mixture was subsequently chilled at −20 °C for 30 min. Post-incubation, samples were centrifuged (13,000× *g*, 4 °C, 15 min). The resulting supernatant was then dried beneath a mild nitrogen flow employing a JXDC-20 evaporator (Shanghai Jingxin Industrial Development Co., Ltd, Shanghai, China). For reconstitution, the dried pellet was dissolved in 100 μL of an acetonitrile/water mixture (1:1, *v*/*v*) (without additional internal standards, as they were incorporated during the initial extraction phase), followed by brief sonication (5 min, 5 °C). A secondary centrifugation step was then applied (13,000× *g*, 4 °C, 10 min). The clarified supernatant was loaded into autosampler vials prior to LC-MS/MS evaluation.

We created a composite quality control (QC) pool by mixing identical aliquots from every experimental specimen. This QC mixture underwent the exact preparation protocol used for test samples and injected regularly during the LC-MS/MS run to assess and correct for instrumental drift and analytical variability. Metabolite analysis profiling relied on a Thermo Vanquish Horizon UHPLC unit integrated with a Q-Exactive HF-X mass spectrometer (Thermo Scientific, Waltham, MA, USA; Majorbio Bio-Pharm Technology Co., Ltd., Shanghai, China). Analytes were resolved using an ACQUITY HSS T3 column (100 mm × 2.1 mm i.d., 1.8 μm; Waters, Milford, MA, USA), held at 40 °C, operating at a flow velocity of 0.40 mL/min. Solvent A comprised an aqueous solution of 0.1% formic acid mixed with acetonitrile (95:5, *v*/*v*), while mobile phase B was 0.1% formic acid in acetonitrile: isopropanol: water (47.5:47.5:5, *v*/*v*). For positive ionization, the elution profile began at 0% B (0–3 min), ramping to 20% (3 min), 35% (4.5 min), and peaking at 100% (5 min). This was maintained until 6.3 min, followed by a return to 0% at 6.4 min, maintaining until 8 min. In negative mode, the elution ramped from 0% to 5% (0–1.5 min), to 10% by 2 min, to 30% by 4.5 min, and to 100% by 5 min, holding until 6.3 min before returning to 0% at 6.4 min and maintaining until 8 min.

The UHPLC system (Vanquish Horizon system, Thermo Scientific, Waltham, MA, USA) was interfaced with the Q-Exactive HF-X mass spectrometer fitted with an electrospray ionization interface. Key MS parameters included a source temperature of 400 °C, sheath gas (40 arb) and auxiliary gas (10 arb). The ion-spray voltage was set to −2800 V (negative) and 3500 V (positive) for positive mode. A stepped normalized collision energy of 20–40–60 V was employed. Scan resolutions were set to 70,000 (Full MS) and 17,500 (MS/MS). We acquired data via Data Dependent Acquisition mode, covering a range of 70–1050 *m*/*z*. Raw LC-MS files were processed via Progenesis QI (Waters Corporation, Milford, MA, USA). For metabolite annotation, we queried established databases, primarily the Human Metabolome Database, Metlin, and the proprietary Majorbio Database.

### 2.9. Data Statistics

Statistical evaluations were executed via the SAS suite (v9.21; SAS Institute Inc., Cary, NC, USA), utilizing a one-way analysis of variance (ANOVA) model for plasma lipid metabolism indicators and fatty acid composition-related data. Prior to testing, data normality and variance homogeneity were confirmed using the Shapiro–Wilk method. For pairwise mean comparisons, we applied Duncan’s multiple range test, expressing results as mean values, and the results are presented as means. Significance was established at *p* < 0.05.

## 3. Results

### 3.1. Plasma Lipid Profiles

Table 2 details the impact of the AEE group on the plasma lipid profile were evaluated. Notably, AME supplementation led to a marked decrease in plasma TG levels (*p* < 0.05). Conversely, the treatment did not significantly alter other lipid metabolic markers (*p* > 0.05). Plasma LDL-C displayed a tendency to increase following AME supplementation (0.05 < *p* < 0.10).

### 3.2. Plasma Fatty Composition

Table 3 outlines the distribution of fatty acids within the plasma. Supplementation with AME led to a marked reduction in the levels of C15:0, C16:1, C18:1n-9 c, and C18:3 n-6, alongside a decrease in the overall proportion of monounsaturated fatty acids (MUFA) (*p* < 0.05). In contrast, the AME regimen significantly elevated C18:2 n-6 c concentrations, accompanied by a rise in total polyunsaturated fatty acids (PUFA) (*p* < 0.05).

### 3.3. Average Daily Dry Matter Intake, Lactation Performance and Milk Profile

Table 4 outlines the impact of AME intake on milk constituents. The results indicated that the AEE intervention failed to notably alter ADMI, DMF, DMP, or DML within the herd (*p* > 0.05).

### 3.4. Data Quality Assurance and Metabolic Profiling Evaluation

**Figure 1:**
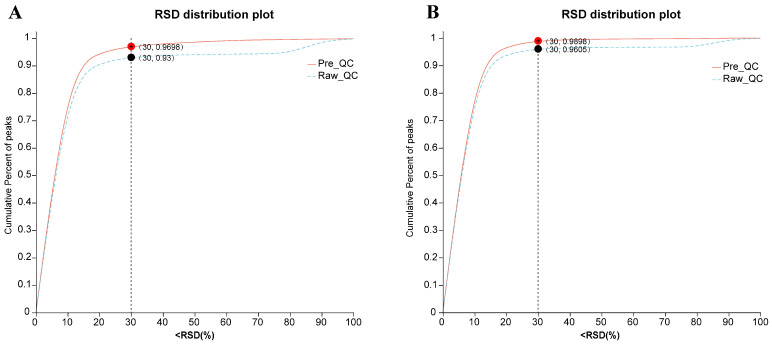
Quality control analysis of milk (**A**) and plasma (**B**) samples. The *x*-axis represents the ratio of the standard deviation to the mean (RSD value, %), and the *y*-axis represents the cumulative proportion of ion peaks. The overall dataset is considered qualified if the cumulative proportion of peaks exceeds 0.7 when the RSD is less than 30%. The dashed line indicates the data before pre-processing, while the solid line indicates the data after pre-processing.

To verify the robustness of our LC-MS/MS instrumentation and data consistency across the dataset, we computed the relative standard deviation (RSD) for all detected metabolic features in QC samples of milk (Figure 1A) and plasma (Figure 1B). As shown in Figure 1A, the analytical system demonstrated excellent stability for the milk samples. The cumulative proportion of ion peaks with an RSD < 30% in both the raw data (Raw_QC) and pre-processed data (Pre_QC) significantly exceeded the standard acceptance threshold of 70%. Notably, the cumulative curve for the pre-processed data (solid line) was consistently positioned above that of the raw data (dashed line), indicating that the data pre-processing step effectively minimized systematic variation and improved data consistency. Similarly, the RSD analysis of the plasma metabolomics data (Figure 1B) exhibited high reproducibility. Using an RSD threshold of <30% as the quality criterion, 96.05% of the ion peaks in the raw data met this standard. Following data pre-processing, this proportion increased to 98.98%. Both values significantly surpass the conventional acceptance threshold of 70%. Furthermore, the upward shift of the pre-processed data curve relative to the raw data confirms the effectiveness of the processing procedure in reducing variation. In conclusion, these results demonstrate the exceptional quality and reliability of the dataset, validating its suitability for subsequent multivariate statistical analysis.

### 3.5. Metabolomic Analysis of Plasma

**Figure 2:**
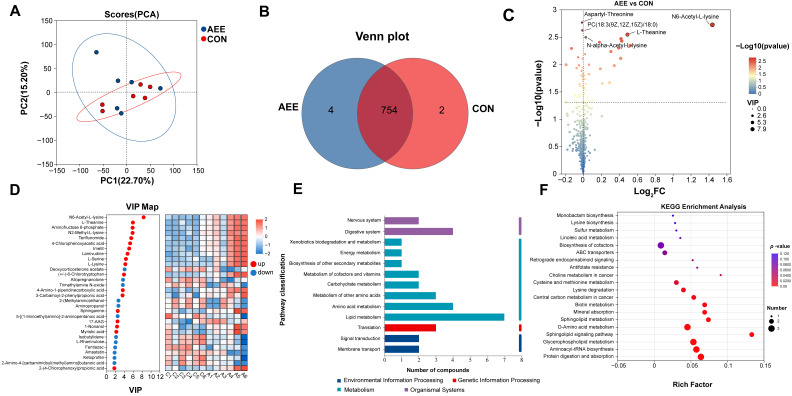
Plasma metabolomics analysis. (**A**) PCA score plot illustrating sample clustering and similarity. (**B**) Venn diagram showing the number of shared and unique metabolites among groups. (**C**) Volcano plot of differential metabolites. The X- and Y-axes represent fold change and statistical significance, respectively. Dot size corresponds to VIP values, indicating up- (**right**) and down-regulated (**left**) metabolites. (**D**) VIP score analysis identifying key discriminatory metabolites. The left panel ranks metabolites by VIP score, while the right panel displays a heatmap of their relative expression levels across samples. (**E**) KEGG functional pathway. The *y*-axis represents the secondary classifications of KEGG pathways, and the *x*-axis indicates the number of metabolites annotated to each pathway. (**F**) KEGG enrichment analysis. The *x*-axis represents the enrichment factor, and the *y*-axis represents the KEGG pathways. The size of the bubbles indicates the number of metabolites enriched in each pathway, while the color of the bubbles represents the significance of enrichment (*p*-value). The cows in the CON group were fed only the basal diet; the cows in the AEE group were fed the basal diet with 54 g/d of *Allium mongolicum* Regel ethanol extract.

Figure 2 illustrates the findings from our plasma metabolomic profiling. Based on the PCA model (Figure 2A), principal component 1 (PC1) accounted for 22.7% of the dataset variability, while PC2 explained 15.2%. Samples from the CON group clustered tightly, whereas those from the AEE group were more dispersed, indicating that supplementation with AME induced a distinct shift in the overall plasma metabolic profile. The Venn diagram analysis (Figure 2B) showed that 4 metabolites were exclusive to the AEE cohort, whereas 2 were specific to the CON cohort, while 754 plasma metabolites were shared between the two groups.

**Figure 3:**
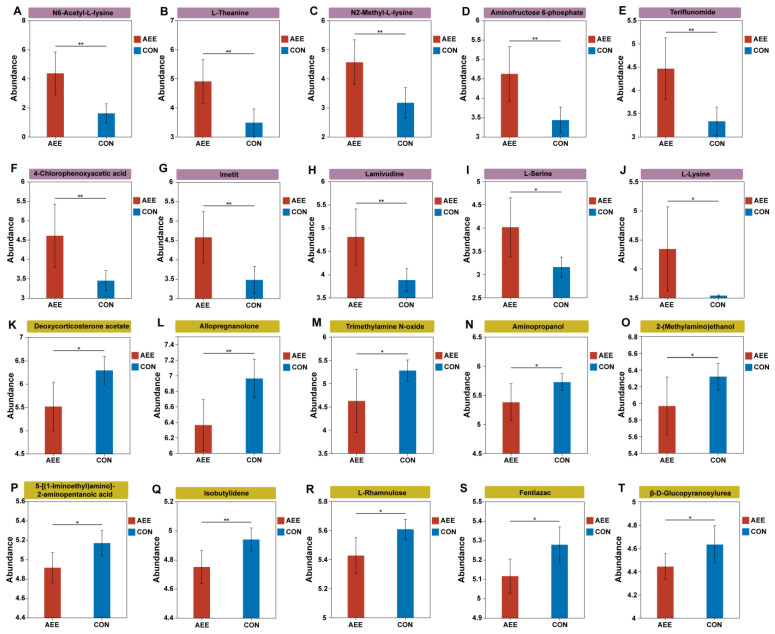
Bar plots of differential metabolites in plasma (TOP 20). *X*-axis: groups; *Y*-axis: MS intensity. Error bars indicate mean ± SD. Purple shading indicates upregulated metabolites, while yellow shading indicates downregulated metabolites. CON = the cows were fed only the basal diet; AEE = the cows were fed the basal diet with an additional 54 g/d per cow of *Allium mongolicum* Regel ethanol extract. Asterisks indicate significant differences: * 0.01 < *p* ≤ 0.05, ** 0.001 < *p* ≤ 0.01, respectively.

The analysis of differential metabolites (Figure 2C) and VIP scores (Figure 2D) identified 46 distinct metabolites separating the AEE and CON cohorts. Relative to the CON group, 26 compounds exhibited increased abundance, whereas 20 were reduced following the AME intervention. The top 10 up-regulated differential metabolites were N6-Acetyl-L-lysine (Figure 3A), L-Theanine (Figure 3B), N2-Methyl-L-lysine (Figure 3C), Aminofructose 6-phosphate (Figure 3D), Teriflunomide (Figure 3E), 4-Chlorophenoxyacetic acid (Figure 3F), lmetit (Figure 3G), Lamivudine (Figure 3H), L-Serine (Figure 3I), and L-Lysine (Figure 3J). The top 10 down-regulated metabolites were Deoxycorticosterone acetate (Figure 3K), Allopregnanolone (Figure 3L), Trimethylamine N-oxide (Figure 3M), Aminopropanol (Figure 3N), 2-(Methylamino) ethanol (Figure 3O), 5-[(1-Iminoethyl) amino]-2-aminopentanoic acid (Figure 3P), Isobutylidene (Figure 3Q), L-Rhamnulose (Figure 3R), Fentiazac (Figure 3S), and β-D-Glucopyranosylurea (Figure 3T).

Regarding the KEGG functional pathways (Figure 2E), a total of 13 pathways were annotated. These specifically included the Nervous system (2); Digestive system (2); Xenobiotics biodegradation and metabolism (1); Energy metabolism (1); Biosynthesis of other secondary metabolites (1); Metabolism of cofactors and vitamins (2); Carbohydrate metabolism (2); Metabolism of other amino acids (3); Amino acid metabolism (4); Lipid metabolism (7); Signal transduction (2); and Membrane transport (2).

Figure 2F illustrates the KEGG pathway enrichment analysis. The results indicate that the metabolites are primarily enriched in pathways including Aminoacyl-tRNA biosynthesis, D-amino acid metabolism, Glycerophospholipid metabolism, Cysteine and methionine metabolism, Protein digestion and absorption, and Biosynthesis of cofactors.

### 3.6. Metabolomic Analysis of Milk

**Figure 4:**
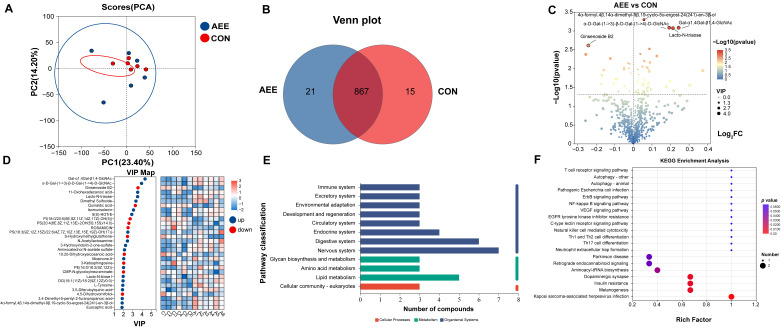
Milk metabolomics analysis. (**A**) PCA score plot illustrating sample clustering and similarity. (**B**) Venn diagram showing the number of shared and unique metabolites among groups. (**C**) Volcano plot of differential metabolites. The X- and Y-axes represent fold change and statistical significance, respectively. Dot size corresponds to VIP values, indicating up- (right) and down-regulated (left) metabolites. (**D**) VIP score analysis identifying key discriminatory metabolites. The left panel ranks metabolites by VIP score, while the right panel displays a heatmap of their relative expression levels across samples. (**E**) KEGG functional pathway. The *y*-axis represents the secondary classifications of KEGG pathways, and the *x*-axis indicates the number of metabolites annotated to each pathway. (**F**) KEGG enrichment analysis. The *x*-axis represents the enrichment factor, and the *y*-axis represents the KEGG pathways. The size of the bubbles indicates the number of metabolites enriched in each pathway, while the color of the bubbles represents the significance of enrichment (*p*-value). The cows in the CON group were fed only the basal diet; the cows in the AEE group were fed the basal diet with 54 g/d of *Allium mongolicum* Regel ethanol extract.

Figure 4 illustrates the findings from our milk metabolomic profiling. As depicted in the PCA plot (Figure 4A), principal PC1 captured 23.4% of the overall dataset variation, while PC2 explained 14.2%. Samples from the CON group clustered tightly, whereas those from the AEE group were relatively dispersed, indicating that the dietary supplementation of AME induced overall alterations in the milk metabolite profile. The Venn diagram analysis (Figure 4B) identified 21 compounds exclusive to the AEE cohort and 15 specifics to the CON cohort, alongside 867 features shared across both sets.

**Figure 5:**
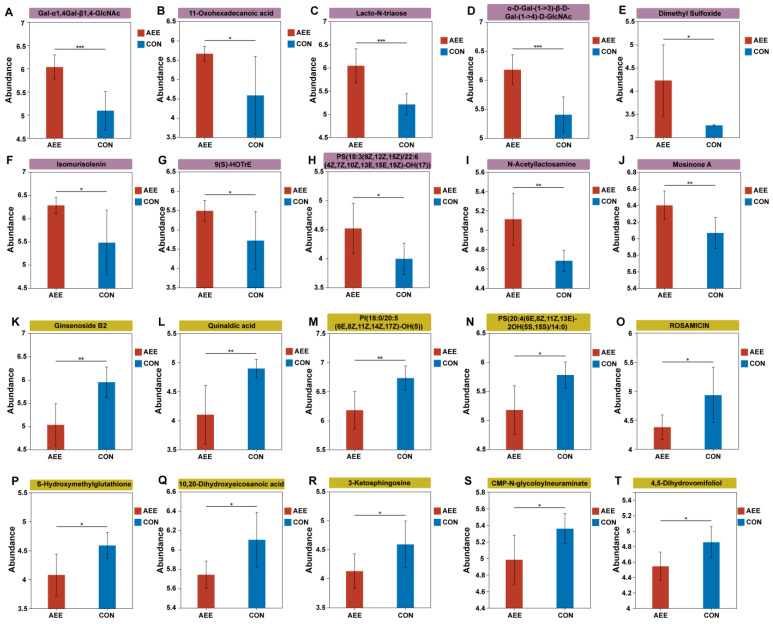
Bar plots of differential metabolites in milk (TOP 20). *X*-axis: groups; *Y*-axis: MS intensity. Error bars indicate mean ± SD. Purple shading indicates upregulated metabolites, while yellow shading indicates downregulated metabolites. CON = the cows were fed only the basal diet; AEE = the cows were fed the basal diet with 54 g/d of *Allium mongolicum* Regel ethanol extract. Asterisks indicate significant differences: * 0.01 < *p* ≤ 0.05, ** 0.001 < *p* ≤ 0.01, *** *p* ≤ 0.001, respectively.

Evaluation of metabolic disparities (Figure 4C) and VIP scores (Figure 4F) highlighted 59 distinct features separating the AEE and CON cohorts. Relative to the CON group, the AME intervention resulted in the enrichment of 42 and depletion of 17 compounds. The top 10 upregulated differential metabolites were Gal-α1,4Gal-β1,4-GlcNAc (Figure 5A), 11-Oxohexadecanoic acid (Figure 5B), Lacto-N-triaose (Figure 5C), α-D-Gal-(1->3)-β-D-Gal-(1->4)-D-GlcNAc (Figure 5D), Dimethyl Sulfoxide (Figure 5E), Isomurisolenin (Figure 5F), 9(S)-HOTrE (Figure 5G), PS(18:3(9Z,12Z,15Z)/22:6(4Z,7Z,10Z,13E,15E,19Z)-OH(17)) (Figure 5H), N-Acetyllactosamine (Figure 5I), and Mosinone A (Figure 5J). The top 10 down regulated metabolites included Ginsenoside B2 (Figure 5K), Quinaldic acid (Figure 5L), PI (18:0/20:5(6E,8Z,11Z,14Z,17Z)-OH (5)) (Figure 5M), PS (20:4(6E,8Z,11Z,13E)-2OH(5S,15S)/14:0) (Figure 5N), Rosamicin (Figure 5O), S-Hydroxymethylglutathione (Figure 5P), 10,20-Dihydroxyeicosanoic acid (Figure 5Q), 3-Ketosphingosine (Figure 5R), CMP-N-glycoloylneuraminate (Figure 5S), and 4,5-Dihydrovomifoliol (Figure 5T).

Regarding the KEGG functional pathways (Figure 4E), a total of 12 pathways were annotated. These specifically included the Immune system (3), Excretory system (3), Environmental adaptation (3), Development and regeneration (3), Circulatory system (3), Endocrine system (4), Digestive system (6), Nervous system (7), Glycan biosynthesis and metabolism (3), Amino acid metabolism (3), Lipid metabolism (5), and Cellular community—eukaryotes (3).

Figure 4F illustrates the KEGG pathway enrichment analysis. The results indicate that the metabolites are primarily enriched in pathways including Parkinson disease, Retrograde endocannabinoid signaling, Aminoacyl-tRNA biosynthesis, Dopaminergic synapse, Insulin resistance, and Melanogenesis.

### 3.7. Associations Linking TG, LDL-C, with Plasma Metabolites

**Figure 6:**
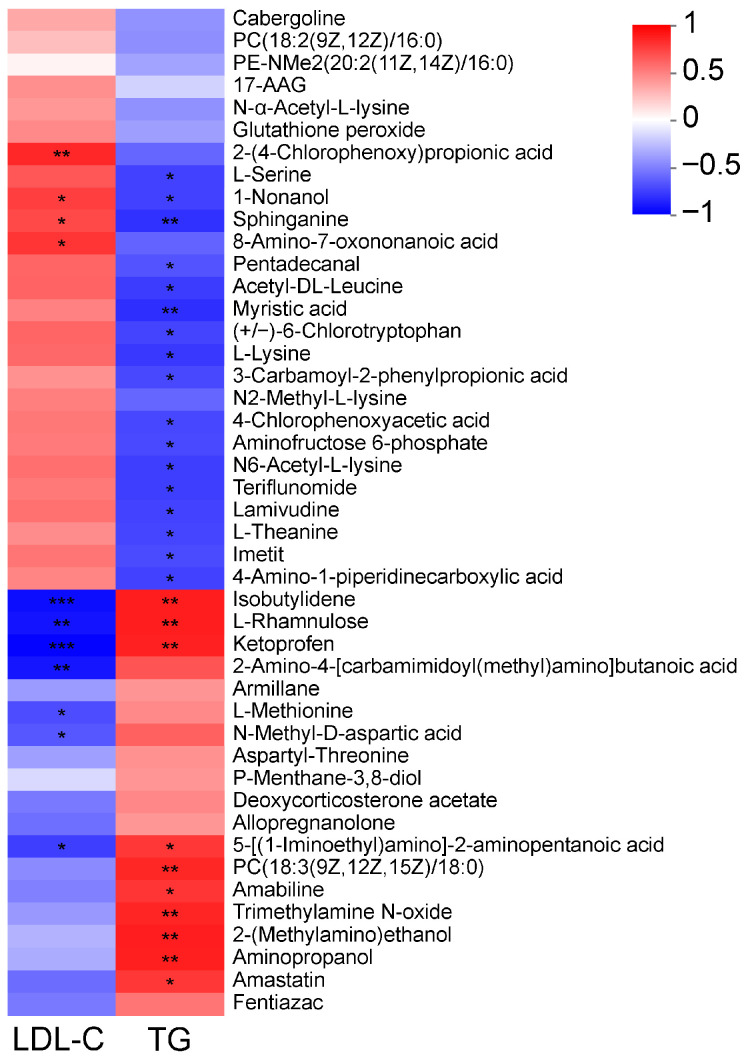
Correlation analysis between plasma metabolites and TG and LDL-C (pearson). The metabolite names are listed on the right, and the associated plasma parameters are listed at the bottom. Each cell represents the correlation between a metabolite and a parameter, with the color indicating the magnitude of the correlation coefficient. Asterisks indicate the level of statistical significance: * 0.01 < *p* ≤ 0.05, ** 0.001 < *p* ≤ 0.01, *** *p* ≤ 0.001, respectively.

Figure 6 visualizes the correlational network among plasma TG, LDL-C, and plasma metabolites. Specifically, our data reveal that LDL-C is positively correlated with 2-(4-chlorophenoxy) propionic acid, Sphinganine, and 8-amino-7-oxononanoic acid, whereas it is negatively correlated with Isobutylidene, L-rhamnulose, Ketoprofen, 2-amino-4-[carbamimidoyl(methyl)amino]butanoic acid, L-methionine, N-methyl-D-aspartic acid, and 5-[(1-iminoethyl)amino]-2-aminopentanoic acid.

Regarding TG, it was negatively correlated with L-serine, Sphinganine, N2-methyl-L-lysine, Aminofructose 6-phosphate, 4-chlorophenoxyacetic acid, N6-acetyl-L-lysine, L-theanine, Imetit, and 4-amino-1-piperidinecarboxylic acid. Conversely, it showed a positive correlation with metabolites including Isobutylidene, L-rhamnulose, Ketoprofen, 5-[(1-iminoethyl) amino]-2-aminopentanoic acid, PC (18:3(9Z,12Z,15Z)/18:0), Trimethylamine N-oxide, 2-(methylamino)ethanol, Aminopropanol, and Amastatin.

### 3.8. Correlation Analysis Between TG, LDL-C and Milk Metabolites

**Figure 7:**
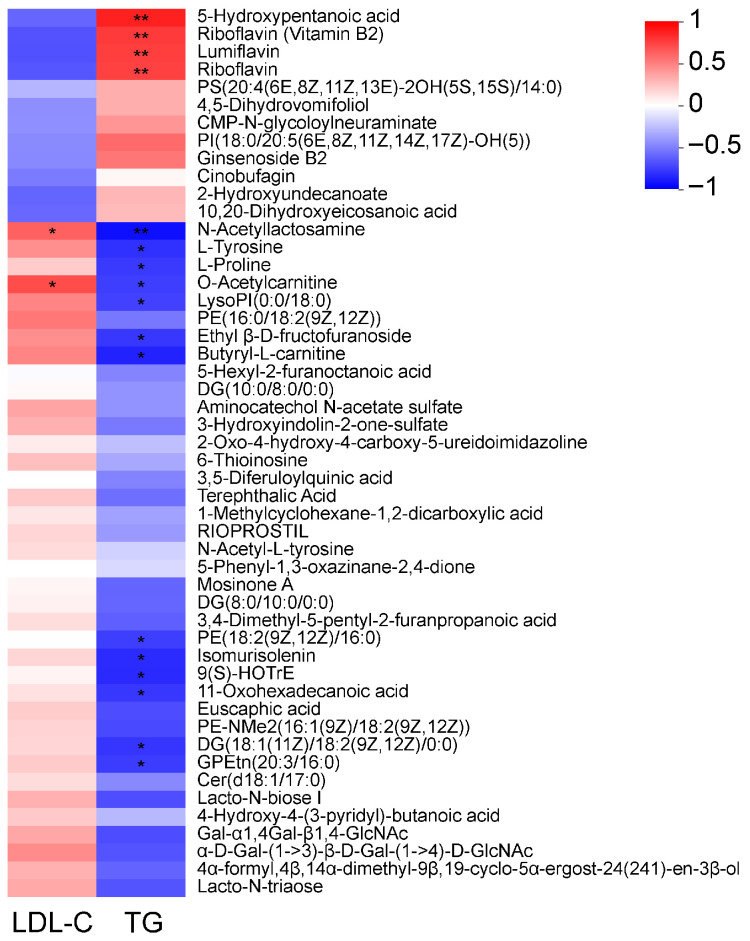
Correlation analysis between milk metabolites and plasma TG, LDL-C (Pearson). The metabolite names are listed on the right, and the associated plasma parameters are listed at the bottom. Each cell represents the correlation between a metabolite and a parameter, with the color indicating the magnitude of the correlation coefficient. Asterisks indicate the level of statistical significance: * 0.01 < *p* ≤ 0.05, ** 0.001 < *p* ≤ 0.01, respectively.

To investigate the potential associations between alterations in milk metabolites and plasma routine parameters, a Pearson correlation analysis was conducted (Figure 7). LDL-C was only positively correlated with N-acetyllactosamine and O-acetylcarnitine. TG was positively correlated with 5-hydroxypentanoic acid, Riboflavin (vitamin B2), Lumiflavin, and Riboflavin, while it was negatively correlated with metabolites such as N-acetyllactosamine, L-tyrosine, L-proline, O-acetylcarnitine, LysoPI (0:0/18:0), Butyryl-L-carnitine, 9(S)-HOTrE, and Isomurisolenin.

## 4. Discussion

### 4.1. Regulatory Impact of Allium Mongolicum Regel Ethanol Extract Intake Regarding Bovine Plasma Lipid Metabolism Alongside Fatty Acid Composition

Plasma triglyceride concentration reflects the efficiency of lipid turnover and partitioning [20]. Typically, plasma TG is primarily packaged within very low-density lipoprotein (VLDL) and chylomicrons, which are trafficked to extrahepatic tissues [21]. During the periparturient period or peak lactation, the hepatic re-esterification of fatty acids operates under high metabolic load, and impaired VLDL secretion can readily precipitate hepatic lipidosis [22,23]. Circulating levels of LDL-C and HDL-C mirror the equilibrium between hepatic cholesterol export to peripheral tissues and reverse cholesterol transport [22,24]. Evidence indicates that subsequent to the development of bovine ketosis, both LDL-C and HDL-C typically decline significantly, potentially attributable to compromised hepatic synthetic capacity and reduced cholesteryl ester transferase activity [25].

In the present study, dietary AME supplementation exerted no significant effects on these indices. This suggests that under the experimental conditions, AEE group did not disrupt normal lipid transport or hepatic metabolism, demonstrating favorable metabolic compatibility without inducing lipid transport dysfunction or hepatic lipid accumulation. Zhao et al. [11] observed that administering 2.8 g/day AME to small-tailed Han sheep markedly lowered plasma TG as well as CHOL concentrations. This discrepancy may be attributed to multiple factors, including physiological state differences, energy requirements, AME dosage, and basal diet composition. In the current study, FFA concentrations following AME supplementation remained unaltered and consistently below the risk threshold (>0.4 mmol/L) [26], indicating that AME did not induce aberrant lipid mobilization, and that energy metabolism likely relied predominantly on dietary nutrient supply rather than adipose tissue mobilization. The stability of β-HB concentrations further demonstrated that AME did not induce abnormal hepatic ketogenesis. Ketosis stands as a leading metabolic condition afflicting periparturient dairy cattle, with clinical diagnosis established when β-HB > 1.2 mmol/L [27,28]. In this study, β-HB concentrations following AME supplementation remained substantially below the clinical diagnostic threshold, suggesting that AME functions as a mild metabolic modulator capable of regulating milk quality without elevating metabolic disorder risk.

Bovine circulating fatty acid signatures serve as an essential indicator reflecting both ruminal metabolism of dietary lipids and host lipid metabolic status [29]. Our current findings reveal that dietary AME markedly elevated circulating fractions comprising C18:2 n-6 c alongside overall PUFA within plasma. Upon ingestion by ruminants, polyunsaturated lipids typically undergo extensive ruminal biohydrogenation, ultimately converting to C18:0 through microbial action [30,31]. Because the current study did not assess specific ruminal parameters, the precise mechanisms driving the observed elevation of plasma linoleic acid remain undetermined. However, substantial evidence demonstrates that secondary metabolites abundant in plant extracts can modulate ruminal microbial communities, particularly by suppressing the function of crucial bacterial genera responsible for biohydrogenation [16,32]. Previous in vitro fermentation research conducted by Wang et al. [16] revealed that AME reduced the prevalence of *Ruminococcus albus*, a bacterium actively involved in hydrogenation. If similar microbial shifts occurred in vivo, they could theoretically enable greater quantities of intact polyunsaturated fatty acids to bypass the rumen and enter systemic circulation. Nevertheless, without direct ruminal data, attributing the improved host fatty acid composition to attenuated biohydrogenation remains speculative and necessitates targeted investigation in future studies.

### 4.2. Remodeling Impact of Allium Mongolicum Regel Ethanol Extract Inclusion upon Plasma Metabolome

Plasma metabolomic analysis revealed upregulation of multiple amino acid-related metabolites, particularly L-serine, alongside protein degradation derivatives such as N6-acetyl-L-lysine and N2-methyl-L-lysine of acetylated and methylated proteins, respectively [33]. Their elevation may reflect enhanced protein turnover or altered lysine metabolic flux induced by AME. This could be associated with the high protein synthesis demands during lactation, although the specific mechanisms require further validation through transcriptomic or proteomic approaches. The increase in L-serine may be related to enhanced one-carbon metabolism and phospholipid synthesis, which are essential for maintaining mammary cell membrane integrity and lactation function. Notably, plasma L-theanine concentration was elevated. Classified as a non-proteinogenic amino acid, L-theanine exhibits anti-stress and neuroprotective properties [34]. Although this compound naturally originates primarily from tea plants, animals can synthesize small amounts through the combination of glutamate and ethylamine. The elevation of L-theanine suggests that AME may indirectly promote endogenous L-theanine synthesis by regulating amino acid metabolism or providing precursor substances, potentially alleviating stress status in dairy cows.

Trimethylamine N-oxide (TMAO) functions as a microbiota-generated byproduct originating from choline, betaine, and the catabolism of carnitine [35], which is primarily produced by ruminal microbiota in ruminants [36]. The observed reduction in plasma TMAO likely reflects AME-mediated modulation of the ruminal microbial community [16]. Notably, this reduction, alongside decreased milk quinaldic acid, suggests coordinated regulatory effects of AME, potentially indicating an optimized ruminal microbial composition and an ameliorated inflammatory response in the mammary gland. Furthermore, the reduction in allopregnanolone—a neurosteroid with known anxiolytic effects [37], and the elevation of L-theanine point towards a modulation of neuroendocrine metabolism. While metabolomic profiles alone are insufficient to define physiological stress levels without corresponding neuroendocrine markers, these metabolic shifts imply that AME supplementation may influence stress-related pathways, potentially altering the systemic demand for endogenous sedative metabolites [38,39]. This multi-level stress relief supports optimal lactation by preventing stress-induced suppression of prolactin and reducing oxidative damage in mammary tissue [40], which partially explains the observed changes in milk antioxidant-related metabolites.

Our current KEGG pathway evaluations demonstrated that Lipid metabolism emerged as the dominant enriched category, involving seven differential metabolites, including L-serine, myristic acid, PC (18:3(9Z,12Z,15Z)/18:0), PC (18:2(9Z,12Z)/16:0), Sphinganine, PENMe2 (20:2(11Z,14Z)/16:0), and Allopregnanolone. This metabolic shift was accompanied by a significant reduction in plasma TG and an increasing trend in LDL-C. In dairy cows, this specific combination of physiological and metabolic changes often indicates a more efficient partitioning of lipids.

The significant decrease in plasma TG, coupled with the different regulation of specific Phosphatidylcholines (PC) and upregulation of phosphatidylethanolamine intermediate (PE-NMe2), suggests that AEE mechanistically promotes hepatic lipid export. In high-yielding dairy cows, the liver faces immense metabolic pressure to process non-esterified lipids released from fat depots. Protecting against the onset of hepatic steatosis relies heavily on the efficient packaging and export of VLDL, which require phosphatidylcholines as obligatory surface components [41,42]. The enrichment of PE-NMe2—a direct intermediate driving the de novo production of PC mediated by the phosphatidylethanolamine N-methyltransferase cascade—alongside their fundamental precursor, L-serine, indicates that AME may alleviate the limitation of phospholipid precursors typically observed during peak lactation [41,43]. Although direct hepatic tissue and lipoprotein secretion data were not evaluated in the present study, the dynamic remodeling of PC species and the increased availability of essential lipotropic precursors suggest a potential metabolic pathway. Consequently, we speculate that AEE may support hepatic lipid metabolism by facilitating the efficient packaging of hepatic triglycerides into VLDL. This accelerated lipid export mechanism effectively mitigates lipid accumulation within hepatocytes, thereby safeguarding liver function and enhancing overall hepatic metabolic capacity [43,44].

Concurrently, the increasing trend of LDL-C further substantiates this enhanced systemic lipid turnover. In the bovine metabolic model, intravascular hydrolysis of VLDL-TG by lipoprotein lipase at peripheral sites—notably the udder to support milk lipid formulation—generates LDL particles [45]. Therefore, the combination of lowered circulating TG and an upward trend in LDL-C strongly implies that AME not only boosts hepatic VLDL output but also accelerates the rapid peripheral uptake and utilization of these triglycerides. Furthermore, the modulation of Sphinganine and myristic acid points toward a profound regulation of cellular lipid signaling. Sphinganine, a critical intermediate in sphingolipid biosynthesis, functions critically to uphold the architecture of cell membrane microdomains while regulating inflammatory responses [46,47]. Interestingly, the significant downregulation of allopregnanolone, a bioactive neurosteroid known for its potent stress-mitigating properties, suggests that the AEE-induced shifts in lipid metabolism effectively alleviate the systemic stress burden, thereby reducing the endogenous demand for such stress-response metabolites [48]. Collectively, these molecular changes demonstrate that AEE supplementation exerts a multi-target beneficial effect: it optimizes the lipid transport axis from the liver to peripheral tissues, reinforces cellular structural stability, and improves the metabolic resilience of dairy cows against lactation-induced physiological stress [37,48,49].

### 4.3. Influence of Allium Mongolicum Regel Ethanol Extract Provision Regarding Bovine Lactation Performance Dairy Cows

In this study, although AME supplementation failed to induce statistically significant alterations concerning overall production volume, along with daily yields of lipid, crude protein, and carbohydrate fractions, these parameters showed slight numerical increases, suggesting that AME may have potential to improve milk composition.

This potential trend could be attributed to the modulatory capacity inherent to bioactive constituents present within AMR, such as flavonoids and polysaccharides, on rumen microbiota. Zhao et al. [50] found in lambs that incorporating 2.8 g/d of AME into their ration diminished the abundance of *Bacteroidetes* and *Prevotella*, while increasing *Firmicutes* abundance. Furthermore, *Prevotella* showed positive correlations with MUFA, revealing the possibility that AME indirectly regulates lipid metabolism through modulating rumen microbiota. However, it should be noted that lambs and dairy cows differ in rumen microbial composition and metabolic characteristics, and whether this mechanism applies to dairy cows requires further verification.

Additionally, the mild promoting effect of AME on milk composition may also be associated with its antioxidant properties. Research by Ding et al. [9] indicated that while AME failed to significantly alter glutathione peroxidase (GSH-Px) activity in lambs, an increasing trend was observed. Wang et al. [10] showed that 15 g/d AMR supplementation increased total antioxidant capacity and catalase (CAT) content in sheep, with GSH-Px also showing an upward trend. Considering that oxidative stress can block milk fat synthesis by suppressing the mammary *NFE2L2* antioxidant pathway and *ACACA* and *FASN* gene expression [51], we speculate that the antioxidant effect of AME may alleviate oxidative stress and create a more favorable physiological environment for milk synthesis.

Notably, the results of this study are similar to other plant-derived additive studies. Khurana et al. [52] documented that incorporating garlic extract into the diet failed to significantly alter lipid and crude protein fractions within lactating bovines but showed slight numerical increases. Bayat et al. [53] also found that plant oil supplementation exerted negligible statistical impact upon production volume and overall secretion profiles within lactating cattle. Collectively, this body of evidence implies that the efficacy of plant-derived additives regarding lactational outcomes is likely dictated by diverse variables, notably feeding duration alongside basal diet composition.

### 4.4. Remodeling Modulatory Influence of Allium Mongolicum Regel Ethanol Extract Inclusion upon Milk Metabolome

Although the addition of AME had no effect on milk composition, our investigation into milk metabolites revealed changes in polysaccharide and oligosaccharide metabolites. Polysaccharides and oligosaccharides play pivotal roles in promoting beneficial microbial colonization and immune modulation in the intestinal tract of young animals. Gao et al. [54] observed that enriching rations with mannan-oligosaccharides elevated serum concentrations of interleukin-10, superoxide dismutase, and GSH-Px in weaned piglets, enhanced tight junction protein expression and intestinal immunological capacity, while subsequently lowering diarrheal occurrences. Paralleling these results [55], dietary xylo-oligosaccharide supplementation improved serum total antioxidant capacity and CAT levels in weaned piglets, while significantly upregulating ileal mRNA expression of interleukin-1β and Interferon. In ruminants, milk oligosaccharides are similarly critical for intestinal development in neonates [56]. In the present study, differentially upregulated metabolites in milk exhibited diverse complex glycan structures, including Gal-α1,4Gal-β1,4-GlcNAc, among others. These studies revealed that AME modulates mammary gland metabolism, enhancing the biosynthetic capacity for functional oligosaccharides possessing prebiotic characteristics, thereby potentially enhancing the nutritional value of milk for calves.

KEGG functional enrichment analysis revealed that the AEE group predominantly upregulated lipid metabolic pathways, including 9 (S)-HOTrE. Although total milk fat content exhibited minimal alteration, AME modified the oxidative status and compositional profile of fatty acids. 9 (S)-HOTrE is a metabolic derivative of α-linolenic acid generated via the lipoxygenase pathway, classified as an oxidized lipid mediator [57]. In contrast to pro-inflammatory arachidonic acid metabolites, α-linolenic acid-derived oxidized lipids typically exhibit anti-inflammatory and pro-resolving properties. Evidence demonstrates that n-3 polyunsaturated fatty acid supplementation ameliorates insulin resistance and aberrant lipid mobilization [58], with mechanisms partially attributed to the generation of specialized pro-resolving mediators derived from EPA and DHA, which facilitate eicosanoid synthesis and anti-inflammatory responses [59,60]. This suggests that AME may attenuate ruminal biohydrogenation, enabling enhanced flow of α-linolenic acid to the mammary gland, where it undergoes conversion to bioactive oxidized lipids via mammary lipoxygenase activity, thereby improving the health-promoting attributes of milk fat.

Furthermore, the marked elevation of Lacto-N-triaose following AME supplementation represents a particularly intriguing metabolic alteration. Lacto-N-triaose, an analog of human milk oligosaccharides, has been extensively validated for its capacity to maintain intestinal barrier integrity [56]. It can be selectively utilized by beneficial bacteria such as *Bacteroides* spp. and *Bifidobacterium* spp., functioning as a prebiotic in the intestinal tract of young animals [61]. The increase in Lacto-N-triaose may be associated with AME-mediated modulation of ruminal microbial metabolism. Previous research has demonstrated that AME alters ruminal microbial composition [16], and certain microbial metabolites, such as short-chain fatty acids may influence mammary glycosyltransferase activity, thereby promoting oligosaccharide synthesis. Additionally, elevated plasma concentrations of Aminofructose 6-phosphate may provide substrates for mammary oligosaccharide biosynthesis. However, the precise mechanisms underlying these effects warrant further investigation.

Several metabolites were downregulated in the AEE group, providing critical insights into improved mammary health in dairy cows. Quinaldic acid, a tryptophan-derived metabolite generated through microbial metabolism, is typically associated with intestinal dysbiosis and inflammatory states [62]. Its reduction in this study suggests that AME may have improved ruminal or intestinal microbial homeostasis, indirectly mitigating inflammatory risk. This interpretation is supported by the concurrent reduction in oxidized phospholipids, including PI (18:0/20:5-OH) and PS (20:4-2OH/14:0). Oxidized phospholipids are typically generated through reactive oxygen species-mediated attack on membrane phospholipids under oxidative stress conditions and serve as biomarkers of cellular damage [63]. Their reduction indicates that AME may have alleviated oxidative injury to mammary cells through antioxidant mechanisms. Additionally, the decrease in S-hydroxymethylglutathione, an intermediate in glutathione-mediated formaldehyde detoxification [64], may reflect reduced endogenous or exogenous formaldehyde generation, or optimized cellular detoxification capacity. The reductions in 10, 20-dihydroxyeicosanoic acid and 3-ketosphingosine further corroborate the anti-inflammatory and antioxidant effects of AME. 10, 20-dihydroxyeicosanoic acid, an ω-oxidation product of arachidonic acid, is elevated under inflammatory conditions [65], while 3-ketosphingosine, an intermediate in sphingolipid metabolism, is associated with cellular stress and apoptosis. The concurrent reduction in these bioactive lipids suggests that AME may coordinately suppress pro-inflammatory and pro-oxidative pathways through multiple mechanisms, thereby reducing the inflammatory and oxidative burden on mammary tissue.

### 4.5. Correlation Analysis Among Plasma Lipid Metabolites and Milk Metabolome

Integrated analysis of milk and plasma metabolomes, combined with metabolite correlation networks, revealed potential mechanisms by which AME may coordinately improve milk quality through regulating amino acid metabolism, glycosylation precursor synthesis, and lipid metabolism. The elevation of amino acid modification and degradation products, such as N^6^-acetyl-L-lysine, N^2^-methyl-L-lysine, along with synthesis precursors like L-serine in plasma, could potentially reflect an altered state of amino acid metabolic flux. These shifts might suggest that AME potentially influences amino acid utilization and systemic protein metabolism, which are crucial for supporting the high protein synthesis demands during lactation [66]. The increase in plasma glucose-6-phosphate indicated enhanced substrate supply for glycolysis and the hexosamine pathway [67]. Glucose-6-phosphate can be converted to UDP-N-acetylglucosamine (UDP-GlcNAc) through a series of enzymatic reactions [67]. UDP-GlcNAc serves as a key precursor for the synthesis of glycoproteins and oligosaccharides [68], particularly N-acetyllactosamine-type oligosaccharides, such as lacto-N-tetraose and lacto-N-neotetraose [56], which possess prebiotic activity and promote the growth of beneficial gut bacteria in infants. This may provide sufficient substrate for mammary synthesis of prebiotic oligosaccharides.

Based on the correlation analysis, we established that AME is associated with the modulation of lipid metabolism from hepatic processing to mammary utilization. Circulating TG and LDL-C may act as critical physiological bridges connecting upstream plasma metabolites with downstream milk metabolites [69]. In the plasma, the glycerophospholipid metabolism pathway was highly enriched, characterized by the upregulation of L-serine. Statistical analyses revealed that plasma TG was negatively associated with L-serine and Sphinganine, whereas LDL-C was positively correlated with Sphinganine. These significant correlations suggest a potential interrelationship: increased availability of phospholipid precursors, such as L-serine and sphinganine, could facilitate the packaging of hepatic lipids into VLDL [70,71]. As the liver efficiently exports these VLDL particles, the accumulation of static TG in the bloodstream may be reduced, while the concentration of LDL-C—the natural byproduct of VLDL hydrolysis—shows a corresponding functional increase [72]. Thus, the altered plasma metabolites appear to be closely linked to profiles of circulating TG and LDL-C [73].

This dynamic shift in blood lipids appears to influence the mammary glands metabolic output. Milk metabolomics revealed the upregulation of short-chain Acylcarnitines, specifically O-acetylcarnitine and butyryl-L-carnitine, which are essential carriers for fatty acid β-oxidation [74,75]. Crucially, our correlation demonstrated that these energy-related milk metabolites were positively associated with LDL-C and negatively associated with plasma TG. This interactive network suggests that the lowered plasma TG and elevated LDL-C might reflect the rapid vascular clearance and active uptake of circulating lipids by the mammary gland [76,77]. As the udder extracts these circulating lipids for energy and milk fat synthesis, the mitochondrial carnitine shuttle is highly activated, leading to the increased secretion of Acylcarnitines into the milk [78,79]. In summary, AEE acts as a systemic metabolic modulator. By enhancing the supply of plasma phospholipid precursors, it alters the systemic pool of TG and LDL-C. It partitions more energy substrates towards the udder, linking hepatic lipid export with mammary lactational performance [80,81]. While this lipid redistribution effectively supports milk synthesis in the short term, further long-term evaluations are warranted to fully understand the broader physiological implications of elevated LDL-C on the overall metabolic homeostasis of the lambs.

### 4.6. Limitation

Several limitations should be considered when interpreting the results of this study. First, the relatively small sample size may limit the statistical power to detect subtle differences in certain metabolic parameters, although the use of metabolomics provided a deep, multi-dimensional view of the metabolic shifts induced by AME. Second, the study did not directly measure rumen fermentation parameters in the dairy cows; therefore, our discussion regarding the modulation of ruminal biohydrogenation by AME remains a hypothesis based on prior in vitro findings, future research should integrate in vivo rumen sampling to confirm these mechanisms. Finally, the short duration of the supplementation trial (28 d) means that our findings only reflect the metabolic impact during a specific window of lactation. Further long-term studies are necessary to evaluate the sustained effects of AME on dairy cow performance, potential cumulative health benefits, and any long-term metabolic adaptations.

## 5. Conclusions

In conclusion, *Allium mongolicum* Regel ethanol extract supplementation significantly alters the metabolomic profiles of dairy cows. It concurrently modulates plasma amino acid and lipid metabolism alongside shifts in milk saccharides and lipid metabolites. These findings highlight a coordinated systemic response, suggesting that *Allium mongolicum* Regel ethanol extract-induced changes in systemic blood metabolism are closely associated with variations in milk composition.

## Figures and Tables

**Table 1:** The ingredient profile and nutritional makeup of the standard ration (DM, %).

Items	Content
Ingredients	
Corn Silage	51.19
Alfalfa	7.28
Wild Oat Herb	1.14
Whole Cottonseed	3.41
Beet Slices	4.55
Corn	15.25
Soybean Meal	7.27
Cottonseed Meal	1.83
Wheat	1.43
Molasses	1.37
Glucose Powder	0.45
Cottonseed Protein Powder	1.58
Fat Powder	0.90
Mycotoxin Binder	0.12
NaHCO_3_	0.18
Yeast Culture	0.30
Premix ^1^	1.75
Total	100.00
Nutrient Levels ^2^	
Net Energy for Lactation (MJ/kg)	6.71
Crude Protein	17.84
Ether Extract	2.50
Neutral Detergent Fiber	29.87
Acid Detergent Fiber	16.10
Ash	7.60
Ca	0.66
P	0.28

^1^ The premix supplied the following per kg of diet: 5775 IU of VA, 1400 IU of VD_3_, 14.80 IU of VE, 12.25 mg of Cu, 0.35 mg of Co, 28 mg of Mn, 0.17 mg of Se, 49 mg of Zn, and 0.87 mg of I. ^2^ Net energy for lactation was a calculated value following Ding et al. [9], while the rest were measured values.

**Table 2:** Plasma lipid metabolism parameters in dairy cows (mmol/L).

Item ^1^	Treatments ^2^		SEM	*p*-Value
	CON	AEE		
TG	0.18	0.15	0.01	0.045
CHOL	4.57	5.17	0.48	0.404
LDL-C	2.73	3.67	0.32	0.066
HDL-C	1.78	1.49	0.23	0.385
FFA	0.13	0.12	0.01	0.313

^1^ TG = Triglyceride; CHOL = Cholesterol; LDL-C = Low-density lipoprotein cholesterol; HDL-C = High-density lipoprotein cholesterol; FFA = Free fatty acid. ^2^ CON = the cows were fed only the basal diet; AEE = the cows were fed the basal diet with 54 g/d of *Allium mongolicum* Regel ethanol extract.

**Table 3:** Plasma fatty acid composition in dairy cows (%).

Item ^1^	Treatments ^2^		SEM	*p*-Value
	CON	AEE		
C4:0	1.30	1.21	0.11	0.438
C14:0	0.67	0.61	0.05	0.276
C14:1	0.64	0.58	0.05	0.214
C15:0	0.47	0.39	0.02	0.004
C15:1	0.03	0.05	0.03	0.705
C16:0	10.33	10.31	0.26	0.927
C16:1	2.01	1.56	0.13	0.006
C17:0	0.32	0.31	0.01	0.438
C18:0	9.98	9.88	0.19	0.635
C18:1 n-9 c	4.88	4.35	0.23	0.046
C18:2 n-6 t	0.18	0.11	0.07	0.383
C18:2 n-6 c	53.52	56.30	0.47	<0.001
C18:3 n-6	2.03	1.64	0.11	0.006
C18:3 n-3	3.21	3.09	0.15	0.448
C20:3 n-6	2.57	2.31	0.16	0.136
C22:1	4.33	4.38	0.35	0.887
C23:0	0.66	0.46	0.33	0.680
C20:4	1.55	1.58	0.61	0.960
C22:2	0.04	0.05	0.03	0.798
C24:0	0.03	0.04	0.03	0.750
C20:5	0.21	0.16	0.09	0.584
C24:1	0.35	0.31	0.04	0.351
C22:6	0.48	0.30	0.15	0.265
MUFA	12.43	11.23	0.41	0.015
PUFA	63.80	65.56	0.73	0.037

^1^ MUFA = the sum of monounsaturated fatty acids (C14:1, C15:1, 16:1, C17:1, C18:1n-9 c, C22:1, C24:1); PUFA = the sum of polyunsaturated fatty acids (C18:2 n-6 t, C18:2 n-6 c, C18:3 n-6, C18:3 n-3, C20:3 n-6, C20:4, C22:2, C20:5, C22:6). ^2^ CON = the cows were fed only the basal diet; AEE = the cows were fed the basal diet with an additional 54 g/d per cow of *Allium mongolicum* Regel ethanol extract.

**Table 4:** Average daily matter intake and lactation performance in dairy cows (kg/d).

Item ^1^	Treatments ^2^		SEM	*p*-Value
	CON	AEE		
ADMI	29.55	30.14	0.28	0.345
Milk yield ^3^	37.30	38.43	1.47	0.740
DMF	1.73	1.78	0.13	0.799
DMP	1.37	1.39	0.06	0.764
DML	1.94	2.00	0.09	0.638

^1^ ADMI = average daily dry matter intake; DMF = daily yields of milk fat; DMP = daily yields of milk protein; DML= daily yields of milk lactose. ^2^ CON = the cows were fed only the basal diet; AEE = the cows were fed the basal diet with 54 g/d of *Allium mongolicum* Regel ethanol extract. ^3^ Milk yield data are cited from Duan et al. [18].

## Data Availability

The raw data supporting the conclusions of this article will be made available by the authors on request.
